# Recreational Drug Use in People Living with HIV in Spain: Factors Associated with Drug Use and the Impact on Clinical Outcomes

**DOI:** 10.1007/s10461-021-03271-3

**Published:** 2021-05-01

**Authors:** Vanessa Castro-Granell, Noé Garin, Ángeles Jaén, José Luis Casado, Lorna Leal, Santiago Cenoz, María José Fuster-RuizdeApodaca

**Affiliations:** 1 Doctoral Programme in Pharmacy, Granada University, Granada, Spain; 2 Department of Pharmacy, Hospital Marina Baixa, Av. Alcalde En Jaume Botella Mayor, 7, 03570 Villajoyosa, Alicante Spain; 3 Department of Pharmacy, Hospital Santa Creu i Sant Pau, Universitat Autònoma de Barcelona, Barcelona, Spain; 4 Instituto de Salud Carlos III, Centro de Investigación Biomédica en Red de Salud Mental (CIBERSAM), Madrid, Spain; 5 School of Health Science Blanquerna, Universitat Ramon Llull, Barcelona, Spain; 6 Research Unit, Fundació Docència i Recerca mutua Terrassa. Universidad de Barcelona, Terrassa, Barcelona Spain; 7 Department of Infectious Diseases, Hospital Ramón y Cajal, Madrid, Spain; 8 Infectious Diseases-HIV Unit, Hospital Clínic Barcelona- IDIBAPS, Barcelona, Spain; 9 Medical Department, ViiV Healthcare, Tres Cantos, Madrid Spain; 10 Spanish Interdisciplinary AIDS Society (Sociedad Española Interdisciplinaria del Sida, SEISIDA), Madrid, Spain; 11 Department of Social and Organizational Psychology, Universidad Nacional de Educación a Distancia (UNED), Madrid, Spain

**Keywords:** HIV, Recreational drug use, Antiretroviral therapy, Clinical outcomes, VIH, Drogas recreativas, Terapia antiretroviral, Resultados clínicos

## Abstract

We analysed the impact of recreational drug use (RDU) on different outcomes in people living with HIV (PLHIV). A multicentre retrospective cohort study was performed with two cohorts of PLHIV included: people using recreational drugs (PURD) vs. people not using recreational drugs (PNURD). Overall, 275 PLHIV were included. RDU was associated with men having sex with men (OR 4.14, 95% CI [1.14, 5.19]), previous sexually transmitted infections (OR 4.00, 95% CI [1.97, 8.13]), and current smoking (OR 2.74, 95% CI [1.44, 5.19]). While the CD_4_/CD_8_ ratio increased amongst PNURD during the follow-up year, it decreased amongst PURD (*p* = 0.050). PURD presented lower scores of self-reported and multi-interval antiretroviral adherence (*p* = 0.017, and *p* = 0.006, respectively), emotional well-being (*p* < 0.0001), and regular follow-up (*p* = 0.059), but paid more visits to the emergency unit (*p* = 0.046). RDU worsens clinical, immunological, and mental health outcomes amongst PLHIV.

## Resumen

Analizamos el impacto del consumo de drogas recreativas sobre variables relacionadas con la salud en personas con VIH (PVIH). Estudio multicéntrico retrospectivo con dos cohortes de PVIH: consumidores de drogas recreativas (CDR) y no consumidores (NCDR). Se incluyeron 275 PVIH. El consumo de drogas recreativas se asoció al colectivo de hombres que mantienen sexo con hombres (OR 4.14, IC95% [1.14, 5.19]), a infecciones de transmisión sexual previas (OR = 4.00, IC95% [1.97, 8.13]) y a ser fumador (OR = 2.74, IC95% [1.44, 5.19]). El ratio CD_4_/CD_8_ aumentó entre los NCDR durante el año de seguimiento y disminuyó en los CDR (p = 0.050). Los CDR presentaron peor adherencia al tratamiento antiretroviral medida con dos métodos indirectos (p = 0.017 y p = 0.006, respectivamente), y bienestar emocional (p < 0.0001). Además, visitaron menos al especialista en enfermedades infecciosas (p = 0.059), y más a urgencias (p = 0.046). El consumo de drogas recreativas empeora los resultados clínicos y de salud mental entre las PVIH.

## Background

Advances in knowledge of HIV infection, as well as the emergence of new therapies, have revolutionised the natural history of the infection, which is currently considered a chronic disease with mortality rates similar to those of the general population [1]. This paradigm shift requires the adaptation of approaches to other emerging problems that can have a decisive impact on the management of HIV, including the prevalence of recreational drug use (RDU) which has potential implications for people living with HIV (PLHIV) in Spain [2, 3].

The pattern of illicit drug use in Spain has changed over time in terms of the substances consumed, the scenarios in which they are used, and user profiles. As a result, its impact on people and society as a whole has also changed [4]. People who injected drugs (PWID) in the 1980s and 1990s were often socially marginalised, and had a tenuous connection with the health system [5]. The prevalence of this profile has decreased from 1994 to 2010, reducing from 3.26 to 0.49 PWID per 1000 inhabitants, thanks to the implementation of specific harm reduction policies [6]. In return, the prevalence of drug use in the ludic or recreational environment has increased by about 50% amongst PLHIV, as studies conducted in Spain and elsewhere in Europe have shown [3, 7–11]. These have placed special emphasis on “polyconsumption,” which is especially predominant in men who have sex with men (MSM) with HIV [12, 13].

The literature shows considerable variability in the frequency of use of different illicit drugs [3, 7–10]. According to the largest study conducted in Spain [3], there were four consumption patterns. Two were composed mostly of heterosexuals (HTX), and the other two principally of MSM. Amongst these studies were two well-differentiated polyconsumption profiles: a group of MSM (whose profile was related to recreational drug use) with the highest rate of polyconsumption, who showed an association with an increased risk of sexually transmitted infections (STIs), and a group of predominantly heterosexual (HTX) individuals (whose profile was associated with the use of traditional drugs such as heroin), who showed worse adherence to antiretroviral treatment (ART) and suffered worse health outcomes. The other two remaining groups presented a pattern of moderate drug use regarding both the frequency and diversity of drugs used. As for the clinical impact of illicit drug use, it seems that the pattern associated with PWID presents clear evidence of poor health outcomes [14–17]. However, interest in recreational drugs is relatively recent and less well known in the field of HIV. Studies have focused primarily on the risk of transmission of infectious diseases, mainly STIs, caused by a higher prevalence of risk behaviours associated with consumption [5, 18–26].

Some data suggest that the use of illicit drugs is predictive of poorer HIV outcomes, although the literature shows inconsistent findings [27]. Few studies have analysed the impact of recreational drug use on immunological parameters (CD_4_, CD_8_, and CD_4_/CD_8_ ratio), but there are cross-sectional studies that used self-reported data [28]. Chao et al. did not find evidence that the use of recreational drugs adversely affects CD_4_ or CD_8_ T cell counts in PLHIV, but they did not report polydrug use [27]. Moreover, several studies have focused on the study of traditional drugs (cocaine, heroin, and crack), and have found contradictory results [29–31]. It is difficult to determine the effect of illicit drug use on CD_4_ and CD_8_ cell count levels. Several factors could influence these cell counts, and there is no consensus in the literature about it. Such factors include the nadir CD_4_ [31], time on antiretroviral therapy [31], adherence level [29, 31], changes in ART [32], age [33], type of ART regimen [29], follow-up time [27], history of STIs [27], and smoking [27], among others. Thus, the impact of polyconsumption of recreational drugs on CD_4_ and CD_8_ counts remains unclear.

Besides immunological parameters, illicit drug use could have an impact on other health-related variables. Studies show that RDU can have a negative impact on adherence to ART [9, 26, 34, 35]. However, the results have been variable and controversial [5, 11, 36–41]. The relationship between RDU and the lack of adherence is complex, and includes intentional and unintentional mechanisms. Adherence is closely linked to viral suppression and is therefore considered a clear predictor of ART success and survival [42]. However, the clinical relevance of the impact of adherence on health in PLHIV using recreational drugs remains uncertain. In fact, some studies suggest that RDU in PLHIV does not affect the viral load level [42, 43]. Certain factors might modulate the final impact of adherence on health outcomes; on the one hand, less restrictive adherence cut-off points (around 80–90%) [44], which achieve viral suppression and, on the other hand, the use of simplified ART regimes [45].

Recreational drug use also has an impact on mental health, although it is difficult to establish causality or directionality because they co-occur very frequently [46]. The negative impact of opioid and stimulant use (including methamphetamines) on the mental health dimension of quality of life [47] has been investigated, and it has been found that their use is associated with increased anxiety, depression, and psychosis [48]. Also, anxiety and depression are particularly prevalent in MSM, and there is a strong association with drug use [49]. Both are associated with risky sexual practices, the increased risk of HIV transmission, and the more frequent use of medical services [50–52].

RDU can also have consequences for the health economy. Some authors claim that the situation may deteriorate because of increased hospitalisations and visits to outpatient and emergency centres [52, 53], dose increases or changes in prescription, or the performance of greater numbers of more invasive diagnostic tests [54]. To the best of our knowledge, no Spanish studies have examined this issue in PLHIV.

Thus, it appears that existing evidence of the impact of RDU on variables related to physical and psychological health is limited; most of the studies are cross-sectional, while the majority focus on the increased risk of STIs, are aimed at the study of traditional drugs and report limited data about the impact of polyconsumption on clinical markers. Thus, the present study, through a retrospective cohort design, had the main objective of examining the impact of recreational drug polyconsumption on several health-related variables in a PLHIV cohort in Spain. It specifically aimed to explore: (1) the variables associated with RDU; (2) potential differences in health outcomes between PLHIV people using recreational drugs (PURD) and people not using recreational drugs (PNURD), including their immunological status evolution during the follow-up period; and (3) potential differences in their use of healthcare services and resources. 

## Method

### Design and Sample

The present study is a part of a broader research project that aimed to analyse several aspects of the use of illicit drugs amongst PLHIV in Spain. We previously conducted a qualitative study interviewing 21 PLHIV who used illicit drugs [2], and a second observational cross-sectional ex-post facto study with 1401 PLHIV [3]. The current third study comprised multicentre observational, retrospective cohort research, in which two cohorts of PLHIV were formed: PURD and PNURD.

We estimated a minimum required sample of 222 PLHIV, with 111 in each group (i.e., PURD and PNURD), accepting an alpha risk of 0.05 and a beta risk of 0.2 in a bilateral contrast (and considering a follow-up loss rate of 10%); the aim was to achieve an expected standardised mean difference in ART adherence of the PURD group of 0.395, based on the results of the meta-analysis by Langebeek et al. [55]. This sample size was sufficient to detect small-to-moderate effect size differences in the other variables under investigation. Due to the fact that we have several outcome variables, we chose ART adherence to estimate sample size; there is more scientific evidence as to what the expected differences between groups would be.

**Fig. 1:**
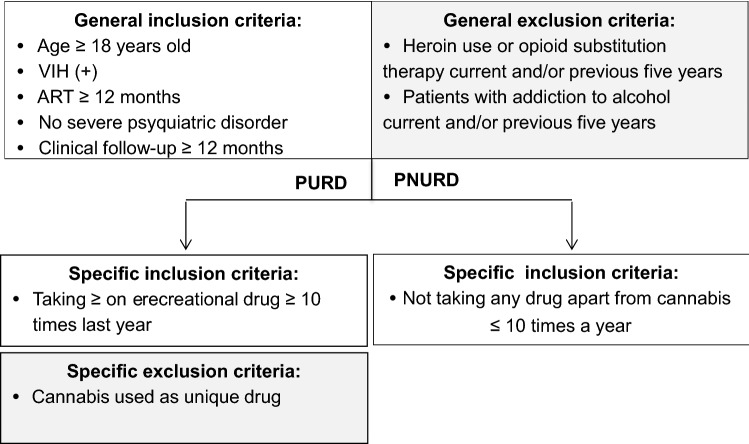
Cohorts of participants, inclusion and exclusion criteria. *PURD* people using recreational drugs, *PNURD* people not using recreational drugs

The general inclusion criteria for both groups were HIV positive diagnosis, age over 18 years, taking ART for at least one year, clinical follow-up of more than 1 year at the centre, and not having any severe psychiatric or cognitive disorder. The specific inclusion criteria for the two cohorts were: PURD (consumption ≥ one drug ≥ 10 times a year, excluding the use of cannabis as a single drug) vs. PNURD (including the consumption of cannabis ≤ 10 times a year). The exclusion criteria for both groups were the consumption of methadone or heroin and dependence on alcohol (current or in the previous 5 years) (Fig. 1).

### Procedure

Data from the study participants were collected from four University Hospitals in three of the most populous Spanish regions (Madrid, Barcelona, and Alicante) between April 2017 and May 2018. Two cohorts of PLHIV were recruited according to the inclusion and exclusion criteria for each group (PURD vs. PNURD) during regular clinical visits. Healthcare providers explained the study’s goals to the participants, requesting their participation and obtained their informed consent. In the same clinical visit, participants responded to a cross-sectional online survey containing the self-reported variables. The survey was self-administered with the support of tablet computers.

**Fig. 2:**
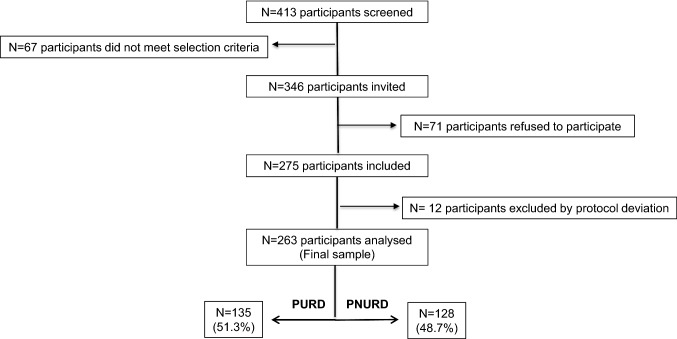
Study sampling. *PURD* people using recreational drugs, *PNURD* people not using recreational drugs

Healthcare providers performed an initial screening of 413 patients during clinical visits. Of them, 67 did not fulfil the selection criteria. Among the remaining 346 patients, 71 refused to participate. Finally, 275 patients were included in the study after they signed the informed consent. The acceptance rate of participation in the study was 79.4%. Out of the 275 PLHIV included in the study (146 PURD and 129 PNURD), 12 participants were excluded due to protocol deviation detected during depurating data. The final analysed sample (*N* = 263) was composed of 135 PURD (51.3%) and 128 PNURD (48.7%) (Fig. 2).

Next, the collaborating researchers collected retrospective clinical data of the previous 12 months from clinical records.

The Ethics Committee of the Hospital Clínico of Valencia approved the research protocol. Written informed consent was obtained from all participants. All of the procedures of the study followed the 1964 Declaration of Helsinki (revised in 1996), as well as the guidelines for good clinical practice. The Spanish AIDS multidisciplinary Society (SEISIDA) coordinated the study.

### Measures

We collected the following retrospective last-12-month clinical data from clinical records: ART adherence (pharmacy refill), CD_4_, CD_4_/CD_8_ ratio, viral load, resistance to and adverse effects of ART, previous clinical conditions, clinical events in the last year, and the use of health services in the last year.

Participants responded to a cross-sectional online survey containing the patient-reported outcome measures. The procedures for designing the survey that was used are described in Fuster-RuizdeApodaca et al. [3]. Variables used in the present analysis were:

#### Use of Illicit Drugs and Other Substances

The survey included items that measured the type of illicit drugs used during the last year, and the frequency and route of consumption. A list of 18 illicit drugs was included.

#### ART Adherence

We used the Questionnaire to Evaluate the Adherence to HIV Therapy (CEAT-VIH) [3, 56]. Higher scores indicate higher treatment adherence.

#### Psychological Well-Being

We used the 12-item validated Spanish version of the General Health Questionnaire (GHQ-12). The higher the score, the worse the psychological health. Normative data in the Western population indicates that scores > 14 in the GHQ-12 are indicative of poor psychological health [57, 58].

The survey also collected several socio-demographic characteristics: age, gender, sexual orientation, education level, employment status, financial resources, and city of residence.

### Data Analysis

We carried out multivariate logistic regression to assess the socio-demographic/clinical factors associated with RDU. We selected the independent variables (covariates) based both on a theoretically based approach (see previous findings in the literature review) and significance at a level of < 0.1 in the bivariate analysis. The categorical covariates included in the model were: sexual orientation (MSM vs. HTX), STIs in the previous year (yes/no), current smoking (yes/no), previous clinical conditions (yes/no), employment status (working vs. not working), and route of HIV transmission (sexual vs. another route). The continuous covariates included were age, level of education, years since HIV diagnosis, monthly incomes, and frequency of drinking alcohol.

Next, we used a linear mixed model for repeated measures over time to analyse the change between the first (baseline) and the last determination during the follow-up year using the following immunological parameters: percentage of CD_4_ and CD_8_ cells/mm^3^ and CD_4_/CD_8_ ratio (within-subject dependent variable) between PURD and PNURD (between-group factor). We included the following covariates in the model: age, time since HIV diagnosis, number or ART changes and ART adherence (pharmacy refill) during the follow-up year.

Finally, to examine the differences in other health-related variables collected once during the follow-up period, we used Student’s *t* or χ^2^, depending on the nature of the data. The analysis was also checked with non-parametric tests; we considered a *p* value < 0.05 to be significant. The analysis was performed using SPSS v.22 software.

## Results

### Characteristics of the Participants

Table 1 shows the socio-demographic, clinical, and epidemiological characteristics of the participants, and differences between PURD and PNURD. The PURD were single, employed, and living in urban areas mostly. They were younger, and had been diagnosed and taking ARV within a shorter time frame than the PNURD. They also contained a higher proportion of MSM.

**Table 1:** Differences between participants who used and who did not use recreational drugs in the variables under study

	Total (*n* = 263)	PURD (*n* = 135, 51.3%)	PNURD (*n* = 128, 48.7%)	*p*-value (Contrast statistic)
Patients’ characteristics				
Age, mean (*SD*; years)	45.79 (10.86)	42.21 (8.24)	49.56 (11.99)	< 0.0001 (*t* = − 5.76)
Gender				0.003 (χ^2^ = 11.45)
Male, % (*n*)	93.5 (246)	98.5 (133)	88.3 (113)	
Female, % (*n*)	6.1 (16)	1.5 (2)	10.9 (14)	
Transgender, % (*n*)	0.4 (1)	0	0.8 (1)	
Sexual orientation				< 0.0001 (χ^2^ = 21.85)
Heterosexual, % (*n*)	12.9 (34)	4.4 (6)	21.9 (28)	
Homosexual, % (*n*)	78.7 (207)	89.6 (121)	67.2 (86)	
Bisexual, % (*n*)	5.7 (15)	3.7 (5)	7.8 (10)	
Others, % (*n*)	2.7 (7)	2.2 (3)	3.1 (4)	
Current relationship				0.034 (χ^2^ = 4.48)
Yes, % (*n*)	40.3 (106)	34.1 (46)	46.9 (60)	
No, % (*n*)	59.7 (57)	65.9 (89)	53.1 (68)	
Educational level				0.080 (χ^2^ = 6.77)
No studies, % (*n*)	1.2 (3)	0	2.4 (3)	
Primary, % (*n*)	17.5 (45)	13.6 (18)	21.6 (27)	
Secondary, % (*n*)	41.6 (107)	42.4 (56)	10.8 (51)	
University degree, % (*n*)	31.7 (102)	43.9 (58)	35.2 (44)	
Work situation				< 0.0001 (χ^2^ = 19.88)
Working, % (*n*)	66.2 (174)	76.3 (103)	55.5 (71)	
Unemployed, % (*n*)	20.2 (53)	17.8 (24)	22.7 (29)	
Retired or disability, % (*n*)	12.5 (33)	4.4 (6)	21.1 (27)	
Other, % (*n*)	1.1 (3)	1.5 (2)	0.8 (1)	
Monthly income^a^	2.77 ± 0.84	2.66 ± 0.82	2.88 ± .84	0.035 (*t* = − 2.12)
Residence				0.015 (χ^2^ = 5.92)
Rural^b^, % (*n*)	4.6 (12)	1.5 (2)	7.8 (10)	
Urban, % (*n*)	94.7 (249)	98.5 (131)	92.2 (118)	< 0.0001 (*t* = − 5.76)
HIV health-related variables				
Time diagnosed, *M* ± *SD* (years)	13.28 ± 7.59	11.70 ± 5.78	14.93 ± 8.80	0.001 (*t* = − 3.37)
Time on ART, *M* ± *SD* (years)	10.67 ± 6.27	9.92 ± 5.66	12.01 ± 7.20	0.014 (*t* = − 2.47)
Previous clinical conditions, % (*n*)	75.7 (199)	82 (105)	69.6 (94)	0.019 (χ^2^ = 5.48)
* M* ± *SD*	2.56 ± 1.80	2.88 ± 2.08	2.20 ± 1.35	0.006 (*t* = -2.76)
Route of transmission				0.051 (χ^2^ = 3.80)
Sexual intercourse, % (*n*)	81.4 (214)	85.9 (116)	76.6 (98)	
Other routes, % (*n*)	18.6 (49)	14.1 (19)	23.4 (30)	
HIV stage, % (*n*)				0.005 (χ^2^ = 21.87)
A1	40.9 (79)	24 (18)	51.7 (61)	
A2	28 (54)	37.3 (28)	22 (26)	
A3	9.3 (18)	10.7 (8)	8.5 (10)	
B1	3.1 (6)	4 (3)	2.5 (3)	
B2	4.1 (8)	2.7 (2)	5.1 (6)	
B3	2.1 (4)	4 (3)	0.8 (1)	
C1	2.6 (5)	2.7 (2)	2.5 (3)	
C2	1 (2)	0	1.7 (2)	
C3	8.8 (17)	14.7 (11)	5.1 (6)	
STIs, % (*n*)	34.6 (91)	51.9 (70)	16.4 (21)	< 0.0001 (χ^2^ = 36.48)
*M* ± *SD*	1.89 ± 1.02	2.04 ± 1.05	1.38 ± 0.74	0.009 (*t* = 2.67)
Current smoker, % (*n*)	49.4 (130)	62.2 (84)	35.9 (46)	< 0.0001 (χ^2^ = 18.16)
Frequency drinking alcohol^c^, *M* ± *SD*	3.98 ± 1.83	4.22 ± 1.68	3.73 ± 1.95	0.032 (*t* = 2.17)

*PURD* people using recreational drugs, *PNURD* people not using recreational drugs

^a^The item ranged from 1 (*none*); 2 *(*≤ *1000 €)*; 3 *(1001–2000 €)*; 4 *(2001–6000 €)* and 5 *(*> *6001 €)*

^b^ “urban” if having more than 10,000 inhabitants

^c^Frequency of drinking alcohol responses ranged from 1 (*never*); 2 *(sometimes a year)*; 3 *(once a month)*; 4 *(sometimes a month)*; 5 *(once a week)*; 6 *(sometimes a week)* and 7 (*daily*)

The most prevalent recreational drugs consumed by the PURD were poppers, cocaine, and cannabis. The average number of different drugs used per participant in the last year was 5.5 (*SD* = 3.2; range 1–14). Injected and rectal administration routes accounted for 10.4% and 9.6% of the cases, respectively (Table 2).

**Table 2:** Type and frequency of recreational drugs used and consumption routes

Drugs	% (N) 100 (135)	*M* ± *SD*^a^ *5.5* ± 3.2
Poppers	81.5 (110)	2.5 ± 1.5
Cocaine (powder)	72.6 (98)	1.9 ± 1.2
Cannabis	63.0 (85)	2.9 ± 2.1
GHB/GBL	57.8 (78)	1.7 ± 1.3
MDMA (pills)	50.4 (68)	1.5 ± 0.8
MDMA (crystal)	45.9 (62)	1.4 ± 0.7
Ketamine	40.7 (55)	1.4 ± 1.0
Mephedrone	40.7 (55)	1.4 ± 0.9
Speed	37.0 (50)	1.4 ± 0.8
Methamphetamine	36.3 (49)	2.1 ± 1.7
Cocaine (base)	5.9 (8)	2.1 ± 1.6
Spice drugs	5.9 (8)	1.5 ± 0.9
LSD	5.2 (7)	1.0 ± 0.0
Mushrooms	3.0 (4)	1.0 ± 0.0
Other hallucinogenic plants	1.5 (2)	1.0 ± 0.0
2C-B nexus	0.7 (1)	1.0

Routes of consumption	% (N)^b^
Sniff	81.5 (110)
Smoke	74.8 (101)
Oral	70.4 (95)
Inhaled	58.5 (79)
Injection	10.4 (14)
Anal	9.6 (13)

^a^Frequency of consumption ranged from 1 (*occasionally during the last 12 months*); 2 (*once a month*); 3 (*sometimes a month*); 4 (*once a week*); 5 (*sometimes a week*) and 6 (*daily*)

^b^Score for each route of consumption was calculated by summing the use of the route in each drug

### Predictors of RDU

The full logistic regression model containing the predictors was statistically significant, χ^2^ (*df* = 11, *n* = 229, PURD = 114, PNURD = 115) = 79.32, *p* < 0.0001, indicating that the model distinguished between PURD and PNURD. The model explained the following percentage of variance: R^2^ = 0.29 (Cox & Snell), 0.390 (Nagelkerke), and 0.25 (Homer & Lemeshow). Based on odds ratio or effect size, MSM compared with HTX, and those who had contracted STIs in the previous year compared with those who had not were around four times more likely to be PURD. Moreover, current smoking was a positive predictor, whereas age was a negative predictor of being in the PURD group (Table 3). The model correctly classified 73.4% of the cases (Table 4).

**Table 3:** Logistic regression analysis of variables related to recreational drug use

Parameter	β	SE β	Wald’s χ^2^ (*df*)	*p*	e^β^ (odds ratio) [95% CI]
Constant	− 1.03	1.35	0.59 (1)	0.355	NA
MSM (vs. HTX)	1.42	0.66	4.68 (1)	0.031	4.14 [1.14, 5.19]
STIs in the previous year (vs. no)	1.39	0.36	14.76 (1)	< 0.0001	4.00 [1.97, 8.13]
Current smoker (vs. no)	1.00	0.33	9.50 (1)	0.002	2.74 [1.44, 5.19]
Frequency of alcohol consumption^a^	0.18	0.09	3.59 (1)	0.058	1.19 [0.99, 1.44]
Age	− 0.05	0.02	7.65 (1)	0.006	0.94 [0.91, 0.98]
Previous clinical conditions (vs. no)	− 0.34	0.39	0.74 (1)	0.390	0.71 [0.33, 1.54]
Income^b^	0.15	0.25	0.36 (1)	0.548	1.16 [0.71, 1.91]
Education level^c^	0.05	0.25	0.04 (1)	0.842	1.05 [0.64, 1.73]
Working (vs. no working)	0.39	0.42	0.85 (1)	0.356	1.48 [0.64, 3.39]
Transmission through sexual intercourse (vs. other routes)	− 0.16	0.47	0.12 (1)	0.731	0.85 [0.34, 2.13]
Years since HIV diagnosis	0.00	0.03	0.03 (1)	0.870	1.00 [0.95, 1.06]
Model fit					

*NA* not applicable, *MSM* men who have sex with men, *HTX* heterosexual, *STIs* sexually transmitted infections

^a^Frequency of alcohol consumption responses ranged from 1 (*never*); 2 *(sometimes a year)*; 3 *(once a month)*; 4 *(sometimes a month)*; 5 *(once a week)*; 6 *(sometimes a week)* and 7 (*daily*)

^b^Monthly income responses ranged from 1 (*none*); 2 *(*≤ *1000 €)*; 3 *(1001–2000 €)*; 4 *(2001–6000 €)* and 5 *(*> *6001 €)*

^c^Level of education responses ranged from 1 (*no studies*); 2 (*primary*); 3 (*secondary*) and 4 (*university degree*)

**Table 4:** Classification table: the observed and predicted frequencies of predictors of drug use estimated by logistic regression

	Predicted		% Corrected^a^
Observed	PURD	PNURD	
PURD	86	28	75.4
PNURD	33	82	71.3
Overall % correct			73.4

*PURD* people using recreational drugs, *PNURD* people not using recreational drugs

^a^Sensitivity = 86/(86 + 28) = 75.4%. Specificity = 82/(33 + 82) = 71.3%. False positive = 33/(33 + 86) = 27.7%. False negative = 28(28 + 82) = 25.4%

### Evolution of Immunological Status During the Follow-Up Year

**Fig. 3:**
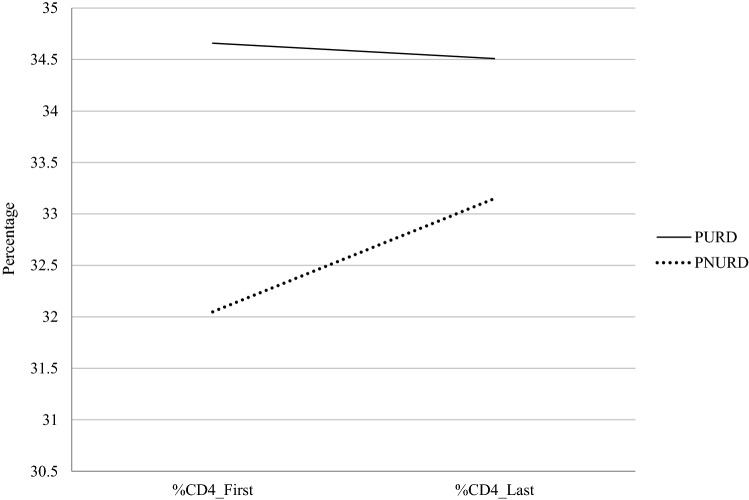
Evolution of CD_4_% cells/mm^3^ in the follow-up year. *Note* The y axis does not start from 0; this is to facilitate visualisation of the graph. *PURD* people using recreational drugs, *PNURD* people not using recreational drugs

The results of the main effects of the linear mixed model for repeated measures did not show a significant difference between the PURD and PNURD in the percentage of CD_4_ (*F* [1, 209] = 2.63, *p* = 0.106). The covariates, number of ART changes and years since HIV diagnosis were marginally related to the participant’s percentage of CD_4_ (F (1, 209) = 3.01, *p* = 0.084 and F (1, 209) = 3.08, p = 0.080, respectively). Also, there was no within subject effects because there was no significant change in CD_4_ cell/mm^3^ between the first and the last determination during the follow-up year (*F* (1, 209) = 0.2, *p* = 0.138). Nevertheless, after controlling the effect of the covariates, the interaction between group membership and the change in the percentage of CD_4_ cells/mm^3^ during the follow-up year was marginally significant (*F* (1, 209) = 3.56, *p* = 0.060) (Fig. 3).

**Fig. 4:**
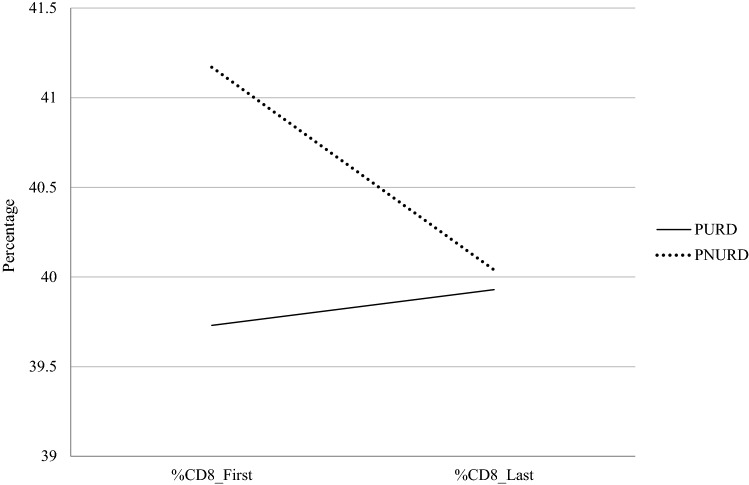
Evolution of CD_8_% cells/mm^3^ in the follow-up year. *Note* The y axis does not start from 0; this is to facilitate visualisation of the graph. *PURD* people using recreational drugs, *PNURD* people not using recreational drugs

Moreover, the results did not show a significant main effect in the percentage of CD_8_ cells/mm^3^ between the PURD and PNURD (*F* (1, 188) = 0.26, *p* = 0.609). Also, none of the covariates showed significant effects in the CD_8_ cells/mm^3^ of the participants. Likewise, there was no within subject effects because there was no significant change in percentage of CD_8_ cells/mm^3^ during the follow-up year (*F* (1, 188) = 2.23, *p* = 0.137). Nevertheless, after controlling for the effect of covariates, there was a significant interaction between group membership and the percentage of CD_8_ cells/mm^3^ (*F* (1, 188) = 4.52, *p* = 0.035). While the percentage of CD_8_ cells/mm^3^ decreased amongst the PNURD, it remained stable in PURD (Fig. 4).

**Fig. 5:**
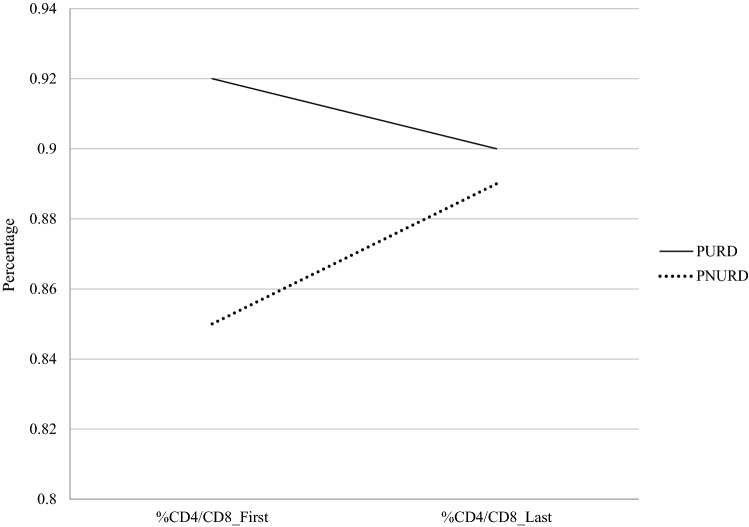
Evolution of the CD_4_/CD_8_ ratio in the follow-up year. *Note* The y axis does not from 0; this is to facilitate visualisation of the graph. *PURD* people using recreational drugs, *PNURD* people not using recreational drugs

Finally, we did not find the main effects between and within subject because there were no significant differences between the PURD and PNURD in the CD_4_/CD_8_ ratio (*F* (1, 188) = 0.45, *p* = 0.503), or in the change in ratio during the follow-up year (*F* (1, 188) = 0.03, *p* = 0.954). Also, none of the covariates showed significant effects in the CD_4_/CD_8_ ratio of the participants. However, we found a significant interaction between intragroup membership and the CD_4_/CD_8_ ratio (*F* (1, 188) = 3.84, *p* = 0.050). While the CD_4_/CD_8_ ratio increased amongst the PNURD during the follow-up year, it decreased amongst the PURD (Fig. 5).

Table 5 presents the marginal means, standard errors and confidence intervals in the immunological variables in both groups.

**Table 5:** Marginal means, standard errors, and 95% confidence intervals in the dependent variables in PURD and PNURD

Dependent variable	Group (Factor)	Measure	Statistics		
			*M*	*SE*	95% CI
CD_4_ cells/mm^3^ (%)	PURD	Baseline	34.66	0.91	32.86, 36.47
	PNURD		32.05	0.86	30.35, 3376
	PURD	Last	34.51	0.86	32.80, 36.22
	PNURD		33.15	0.82	31.53, 34.77
CD_8_ cells/mm^3^ (%)	PURD	Baseline	39.73	1.11	37.54, 41.93
	PNURD		41.17	1.07	39.04, 43.29
	PURD	Last	39.93	1.01	37.93, 41.93
	PNURD		40.04	0.98	38.11, 41.97
CD_4_/CD_8_ ratio	PURD	Baseline	0.92	0.44	0.84, 1.01
	PNURD		0.85	0.04	0.77, 0.93
	PURD	Last	0.90	0.04	0.82, 0.98
	PNURD		0.89	0.04	0.81, 0.97

*PURD* people using recreational drugs, *PNURD* people not using recreational drugs

### Differences in Health Outcomes and Use of Healthcare Services and Resources

Both the PURD and PNURD maintained undetectable viral loads throughout the follow-up year without group differences. However, there was a higher percentage of PURD than PNURD who had experienced changes in their ART regimen during the follow-up year, mainly because of adverse effects and drug-drug interactions. Moreover, a marginally higher number of PURD than PNURD presented non-AIDS related events.

We observed a higher proportion and number of analytical tests performed amongst the PURD. A higher percentage of PURD visited emergency units and a lower percentage visited specialised care units. In addition, PURD participants presented lower scores in the two measures of ART adherence (i.e., self-report measured through a validated questionnaire and multi-interval adherence calculated through pharmacy refills) than PNURD. Finally, the PURD presented poorer psychological well-being than the PNURD (Table 6).

**Table 6:** Differences between PURD and PNURD in health-related variables

HIV health-related variables	PURD (*n* = 135, 51.3%)	PNURD (*n* = 128, 48.7%)	*p*-value (χ^2^)
Viral load undetectable in all tests of the follow-up period, % (*n*)	83.0 (112)	84.4 (108)	0.868 (χ^2^ = 0.09)
Non-AIDS related events, % (*n*)	30.4 (41)	20.3 (26)	0.061(χ^2^ = 3.50)
Opportunistic infection, % (*n*)	4.4 (6)	6.3 (8)	0.514 (χ^2^ = 0.42)
Adverse events, % (*n*)	14.1 (19)	10.9 (14)	0.443 (χ^2^ = 0.58)
Change in ART regimen, % (*n*)	35.6 (48)	21.9% (28)	0.014 (χ^2^ = 5.98)
Resistance tests, % (*n*)	3 (4)	3.9 (5)	0.674 (χ^2^ = 0.17)
Use of healthcare resources			
Diagnostic tests, % (*n*)	64.4 (87)	64.8 (83)	0.946 (χ^2^ = 0.00)
Radiological tests, % (*n*)	45.9 (62)	55.5 (71)	0.122 (χ^2^ = 2.39)
Analytical tests, % (*n*)	26.7 (36)	12.5 (16)	0.004 (χ^2^ = 8.31)
Pathological anatomy tests, % (*n*)	9.6 (13)	13.3 (17)	0.352 (χ^2^ = 0.86)
Surgical procedure tests, % (*n*)	0.7 (1)	3.9 (5)	0.086 (χ^2^ = 2.95)
Exploratory tests, % (n)	0	3.9 (5)	NA
Electromagnetic tests, % (*n*)	4.4 (6)	7.8 (10)	0.253 (χ^2^ = 1.30)
Other tests % (*n*)	3.7 (5)	6.3 (8)	0.341 (χ^2^ = 0.90)
Use of healthcare services			
Primary care visits, % (*n*)	5.9 (8)	1.6 (2)	0.064 (χ^2^ = 3.42)
Specialised care visits, % (*n*)	83.7 (113)	91.4 (117)	0.059 (χ^2^ = 3.55)
Emergency room visits, % (*n)*	61.8 (42)	38.2 (26)	0.046 (χ^2^ = 3.99)
Hospitalisation, % (*n)*	3.7 (5)	6.3 (8)	0.341 (χ^2^ = 0.90)
ART adherence			
Multi-interval adherence (pharmacy refill), *M* ± *SD*	90.9 ± 14.2	94.5 ± 9.2	0.017 (*t* = − 2.40)
Self-reported adherence (CEAT-HIV), *M* ± *SD*	87.8 ± 9.3	90.6 ± 6.7	0.006 (*t* = − 2.74)
Psychological well-being^a^, *M* ± *SD*	12.88.1 ± 6.3	10.62 ± 4.3	0.0001 (*t* = 3.38)

*PURD* people using recreational drugs, *PNURD* people not using recreational drugs, *ART* antiretroviral treatment, *NA* not applicable

^a^The scores ranged from 0 to 36. Means and standard deviations were calculated using the number of patients with presence (yes) in the variable. All mean differences were also tested through non-parametric tests

## Discussion

To the best of our knowledge, the present study is the first to evaluate the association between RDU and clinical variables of physical and psychological health, as well as variables of health economy, in a PLHIV cohort in Spain.

First, we examined the variables associated with RDU in PLHIV. We found that the strongest associations were: being MSM, having suffered STIs during the previous year, and being a regular smoker. The frequency of consumption of alcoholic beverages and being younger were also associated with RDU, albeit at a lower intensity. These results confirm the findings of other studies, where a higher prevalence of RDU amongst those of a younger age [3, 59, 60] and a higher incidence of STIs in the HIV population [3, 61–63] were found. Legal drugs such as alcohol and tobacco are the most commonly used drugs amongst MSM, according to the European MSM Internet Survey [8]. Most studies include alcohol and tobacco when studying the effects of drugs, and show that a high percentage of patients consume recreational drugs and alcohol combined with tobacco [43, 64]. Therefore, it makes sense to think that alcohol or tobacco use is related to RDU.

Following this, we examined differences in health outcomes as a function of using recreational drugs or not. The linear mixed model results did not find significant differences in the percentage of CD_4_, CD_8,_ or CD_4_/CD_8_ between the PURD and PNURD. The epidemiological and clinical variables that characterised the PURDs, such as being HIV positive for a shorter time, being younger, or having fewer clinical antecedents [3], might have influenced the lack of difference in the cross-sectional measures between both groups. The results did not show any differences within-subjects because there were no significant changes in the immune parameters during the follow-up year. However, we found significant interaction effects between the evolution of immune parameters in the follow-up period and group membership. While the PURD group experienced a decrease in the CD_4_/CD_8_ ratio throughout the follow-up, it increased in the PNURD group. This increase was associated with a decrease in the percentage of CD_8_ and an increase in the percentage of CD_4_ during the follow-up year in the PNURD group, whereas the PURD group remained stable both in the evolution of CD_4_ and CD_8_. We found the previous results controlling some covariates which the literature shows could influence immune parameters such as age, time since diagnosis, adherence level, and ART changes [29, 31–33]. However, our results should be interpreted with caution because other potential variables might influence the results. Furthermore, it has been suggested that being MSM could be associated with a change in the CD_4_/CD_8_ ratio because bacterial and viral agents responsible for STIs, which are more common among MSM, may increase immune activation and result in a continuous expansion of the CD_8_ population [32]. The CD_4_/CD_8_ ratio represents a predictor of age-related diseases and non-HIV-associated events (e.g., ischemic heart disease, stroke, and chronic kidney failure [65] and higher mortality [66]. Moreover, CD_4_/CD_8_ ratio recently has been shown to be a more sensitive immune prognostic marker of adverse outcomes than the CD_4_ cell count which frequently normalises with effective ART [32]. Thus, further longitudinal studies that analyse longer follow-up times and more potential covariates that the current research, should deepen the impact of the polyconsumption of recreational drugs on immune parameters.

Additionally, we found that the adherence rate measured by two indirect evaluation methods was lower in the PURD group. Our results are consistent with evidence showing that people who use drugs are more likely to experience adherence problems [9, 26, 34, 35]. However, adherence does not seem to be determinant in the changes we found in the immune markers’ evolution, in agreement with other studies [33]. On the contrary, previous studies found that adherence levels influence the decline of these markers [29, 31]. However, these studies were conducted prior to current ART therapy which determines the less strict adherence to cut-off points [44]. Thus, our sample’s adherence level does not appear to have impacted their virological suppression because a small percentage of participants presented detectable viral loads. In this regard, some studies claim that RDU in PLHIV does not negatively impact the viral load [42, 43], and argue that the most influential aspect in terms of health impact is not specifically drug use itself, but the degree of dependence on drug use. It should be noted that although a small percentage presented a detectable viral load (about 15% of patients), the impact could be transcendent at the level of HIV transmissibility, especially in the MSM group, who are involved in more extreme sexual practices (i.e., chemsex). It is to be hoped that, over time, the difference between the groups will be more pronounced and may have a greater impact on patients’ viral load. According to published studies, adherence remains at more stable levels for the first 24 months of treatment and decreases by 5% every 6 months [42]. Adherence to ART is paramount to controlling HIV infection, and is associated with positive clinical outcomes [67, 68].

The present study also found that the PURD had experienced more ART treatment changes during the follow-up year. Moreover, the linear mixed model results found a marginal association with it and participant’s percentage of CD_4_. According to published studies, ART changes are a predictor of viral suppression failure [69]. The side effects and toxicity associated with treatment and interactions were the main causes of ART changes. Some studies have found a high prevalence of interactions between ART and recreational drugs, in some cases with severe consequences [26]. Thus, close attention should be paid to adequately addressing factors associated with ART changes.

Likewise, we found that the PURD had worse psychological health than the PNURD. Anxiety and depression are two of the most prevalent symptoms amongst PLHIV [48, 49], and both may be at the root and the result of problematic drug use [70]. Drug use is also related to dependence. This is a complex relationship that can cause mental health problems, stigmatisation, and sexual acceptance, amongst others [71].

In our study, a higher proportion of patients visiting emergency units was found amongst the PURDs (1.6 times higher than the PNURD), which did not include patients with severe mental morbidity—according to published studies, this seems to be a determinant of hospital morbidity [51, 72, 73]. We also found a high frequency of emergency visits (175 visits per 100 patients per year), although there were no differences between the PURD and PNURD. This might be explained by the fact that the PNURD had longer medical histories. According to a meta-analysis [53], the combined rate in the studies covered was 151 emergency visits per 100 people per year in the general drug-using population. Our study reflects a high demand for emergency care in the drug consuming PLHIV population. Predictors of risk of hospital emergency services by drug-consuming patients include patients diagnosed with HIV, mental health problems, and polyconsumption [14, 51, 74–79].

Our study has some limitations. Because it is observational, the relationships may be influenced by confounding variables. Although we use a longitudinal design, our findings are limited by the short follow-up period. Furthermore, because of the retrospective study design, we could not collect relevant data that are potentially related to our findings, such as the frequency and number of drugs consumed throughout the follow-up period. Also, some of the data presented come from a self-reported questionnaire, and there was no way to verify the veracity of the information. It is possible that respondents may have attenuated their answers with regard to drug use, which would have led to information bias. However, our results should be interpreted in light of the extensive research conducted. We carried out various types of triangulation, obtaining different types of data through different methodologies. This will have given our conclusions greater validity.

## Conclusions

According to our results, RDU in PLHIV who are in ART treatment has clinical implications for health-related variables, such as immune status, adherence to ART treatment, psychological well-being, and the use of healthcare resources.

The long-term impact of using recreational drugs on PLHIV’s health could be more extensive than our results suggest. Patients belonging to the PURD group presented better health status than the PNURD group, who had a higher rate of associated comorbidities. Thus, interventions are needed to reduce the negative impact of drug use on health. These interventions should provide patients with adequate information on the potential risks related to RDU: drug-drug interactions, adverse effects, and the impact on physical and psychological health. Moreover, appropriate ART is necessary for patients who use drugs, so that changes due to interactions and side effects that may produce resistance and therapeutic failure can be avoided. These actions should preferably target MSMs with HIV and young people who have previously experienced STIs.

## Data Availability

Data are available from https://doi.org/10.6084/m9.figshare.13090142.v1

## Abbreviations

AIDS: Acquired immunodeficiency syndrome
ART: Antiretroviral therapy
HIV: Human immunodeficiency virus
HTX: Heterosexual
MSM: Men who have sex with men
PLHIV: People living with HIV
PWID: People who inject drugs
STIs: Sexually transmitted infections
RDU: Recreational drug use
PURD: People who use recreational drugs
PNURD: People who do not use recreational drugs
